# Transformation and articulation of clinical data to understand students’ clinical reasoning: a scoping review

**DOI:** 10.1186/s12909-025-06644-7

**Published:** 2025-01-12

**Authors:** Marie-France Deschênes, Nicolas Fernandez, Kathleen Lechasseur, Marie-Ève Caty, Busra Meryem Uctu, Yasmine Bouzeghrane, Patrick Lavoie

**Affiliations:** 1 Faculté des sciences infirmières, Université de Montréal, Succ. Centre-Ville, Montréal, C. P. 6128, H3C 3J7 Canada; 2 Centre for Interdisciplinary Research in Rehabilitation of Greater Montreal (CRIR), Montréal, Canada; 3 Department of Family Medicine and Emergency Medicine, Faculty of Medicine, Université de Montréal, Montréal, Canada; 4 Faculté des sciences infirmières, Université Laval, Québec, Canada; 5 Département d’orthophonie, Université du Québec à Trois-Rivières, Trois-Rivières, Canada; 6 Montreal Heart Institute Research Center, Montréal, Canada

**Keywords:** Clinical reasoning, Semantic qualifiers, Discourse, Linguistics, Education, Natural language processing, Scoping review

## Abstract

**Background:**

Despite the importance of effective educational strategies to promote the transformation and articulation of clinical data while teaching and learning clinical reasoning, unanswered questions remain. Understanding how these cognitive operations can be observed and assessed is crucial, particularly considering the rapid growth of artificial intelligence and its integration into health education. A scoping review was conducted to map the literature regarding educational strategies to support transformation and articulation of clinical data, the learning tasks expected of students when exposed to these strategies and methods used to assess individuals’ proficiency

**Methods:**

Based on the Joanna Briggs Institute methodology, the authors searched 5 databases (CINAHL, MEDLINE, EMBASE, PsycINFO and Web of Science), ProQuest Dissertations & Theses electronic database and Google Scholar. The data were synthesized narratively using descriptive statistics.

**Results:**

A total of 38 articles were included in the final synthesis. Most studies were conducted in North America and Europe (*n =* 30, 79%) focused primarily on medical students (*n* = 35, 92%) and mainly used observational (*n* = 17, 45%) or methodological (*n* = 8, 21%) designs. Various educational strategies were identified, the most common were resolution of written or computerized case-based scenarios (*n* = 13; 52%) and simulated or real patient encounters (*n* = 6; 24%). The learning tasks comprised, among others, identifying key findings, translating clinical information, synthesizing cases aloud, and writing a summary statement. Furthermore, the review included assessment methods and rubrics with assessment criteria for clinical data transformation and articulation. The narrative synthesis shows positive results when integrating various educational strategies within clinical reasoning curricula compared to a single strategy used episodically.

**Limitations and conclusions:**

The varying objectives, diversity of educational strategies documented, and heterogeneity of the evaluation tools or rubrics limit our conclusions. However, insights gained will help educators develop effective approaches for teaching clinical reasoning. Additional research is needed to evaluate the impacts of educational strategies aimed at developing skills for the transformation and articulation of clinical data.

**Clinical trial number:**

Not applicable.

**Supplementary Information:**

The online version contains supplementary material available at 10.1186/s12909-025-06644-7.

## Background

Promoting development of health care professionals’ clinical reasoning in education is crucial to ensure safe and effective practice [1, 2]. Clinical reasoning is a complex process that involves identifying relevant patient data, generating and testing clinical hypotheses, determining an intervention plan, and foreseeing a situation’s immediate and later evolution [3, 4]. Clinical reasoning has been included within formal competency frameworks across health professions internationally.

In recent decades, many efforts have been made to develop multiple educational strategies to foster the development of students’ clinical reasoning (e.g., case studies, concept mapping, and simulation) [5]. Methods for assessing clinical reasoning (e.g., objective structured clinical examination (OSCE), oral case presentation, and key-feature exams) have also been implemented [6]. However, educators are often confronted with the complexity of evaluating specific cognitive operations that illustrate the pragmatic exercise of clinical reasoning, especially the activation, organization, and reorganization of professional knowledge [4].

Transforming and articulating clinical data are cognitive operations central to health professionals’ clinical reasoning processes. Transformation of clinical data refers to the process of abstraction or interpretation emerging from “problem representation” [7], an early mental representation of a clinical situation which may include elements from the patient’s history, the findings on physical examination, results of lab tests [7]. This process can be relatively unconscious but could be generally understood as a one-sentence summary statement of the clinical situation [7]. Articulation of clinical data refers to “putting into words” the interpretation of the data of the situation into abstract terms, i.e., medical terminology [7].

These cognitive operations transform the “raw” information expressed in lay terms, to articulate the relevant clinical data using specialized and precise terms [8]. Here is an illustration of the translation and articulation of clinical data from the patient’s lay language to the semantic qualifiers used by health professionals, based on a patient’s clinical history:

Mrs. Ferguson is a 51-year-old woman who has been experiencing difficulty breathing for the past 24 h at rest and without physical effort. When she speaks, she has to stop several times to catch her breath. Sounds resembling bubbles popping are heard on auscultation and are located in both pulmonary bases.

Mrs. Ferguson becomes “female”; 51-year-old becomes “middle-aged”; last 24 h becomes “onset”; difficulty breathing becomes “dyspnea”; bubbles popping becomes “crackles” and both pulmonary bases become “bilateral.” Translated using semantic qualifiers, the story becomes:

A middle-aged female presents with an onset dyspnea at rest and on exertion (e.g., talking) characterized by bilateral crackles in pulmonary bases on auscultation.

In the 1990s, Lemieux and Bordage [9] introduced the concept of quality of health professionals’ discourse as indicators of clinical reasoning mastery, namely its semantic and syntactic components. The semantic component of professional discourse involves semantic qualifiers, which reflect the intelligible organization of knowledge into meaning units [8, 10]. These semantic qualifiers, such as “acute” versus “chronic” pain or “proximal” versus “distal”, serve as building blocks for organizing and indexing knowledge in long-term memory. The syntactic component of professional discourse reflects the richness and structure of knowledge in long-term memory [8, 10]. Discourse may appear scattered or reduced when knowledge is missing or insufficiently developed to understand and respond to a situation effectively. Thus, Bordage et al. [11] proposed a discourse classification system based on the use of semantic qualifiers and syntactic components (synthesis and conciseness related to clinical hypotheses). Appendix A presents these categories, from reduced and scattered discourses to elaborate and compiled discourses. We also present narratives illustrating each category as observed in a nurse-to-physician interaction.

The appropriate transformation of patients’ lay terms into a professional vocabulary (e.g., medical terminology) implies that the learner has well-organized professional knowledge networks that are rich enough to establish connections between data, capture situational nuances, generate clinical hypotheses, and even predict what could happen in the near future [8, 10]. Furthermore, these cognitive operations are vital for ensuring the consistency and efficiency of information exchanges (e.g., handoff, documentation) between professionals, thus contributing to safer practices [7].

Despite the importance of effective educational strategies to promote the transformation and articulation of clinical data while teaching and learning clinical reasoning, unanswered questions remain. For example, are traditional educational teaching strategies (e.g., learning medical terminology, practicing history-taking skills, solving clinical case studies) still relevant? Should they be performed in a logical sequence to enhance the development of clinical reasoning and thus better prepare students for immersive clinical experiences (i.e., clinical rotations)? Is the use of think-aloud feasible and commonly used in health education programs? Do written medical notes provide sufficient material to assess the development of students’ clinical reasoning? Moreover, given the variety of educational strategies, selecting and implementing strategies to solicit cognitive operations of clinical reasoning in health education programs is challenging for educators who must rely on their local resources and consider some situational constraints.

Findings ways to observe and assess transformation and articulation of clinical data is crucial in the current context where artificial intelligence (AI) tools, particularly those modeling and reproducing human language capabilities [12, 13], are being implemented. For example, Maicher et al. [14] developed a system for learners to practice history-taking skills and documentation via natural language conversations with virtual patients. Schaye et al. [15] assessed the clinical reasoning documentation quality of students’ notes using a supervised machine learning model. These AI tools potentially challenge how learning tasks and the cognitive processes of clinical reasoning are learned, taught, and assessed [13]. Thus, understanding how learners’ clinical reasoning can be transformed and articulated in natural language processing is potentially very useful.

Therefore, this scoping review seeks to map educational strategies that support health care students’ articulation and transformation of clinical data into language easily translatable into natural language processing. A secondary objective is to explore the methods used to observe and assess these cognitive operations.

## Methods

Based on the Joanna Briggs Institute guidelines [16], the scoping review uses the Preferred Reporting Items for Systematic Reviews and Meta-Analyses extension of scoping reviews (PRISMA-ScR) [17]. This methodology allows for exploring a broad research question using diverse methods and approaches while retaining systematic and replicable methods [18]. The protocol was registered on the Open Science Framework (https://osf.io/td9p4/) and was published [19]. Since the publication of the protocol and considering the preliminary analysis of the results, we have added one research question, i.e., question 2 below, and circumscribed the targeted context (i.e., the educational context of students in health education programs). These changes were made considering the presence of additional results which were consistent with the educational perspective (question 2) of this review, while the presence of heterogeneous data regarding the research aims prompted us to better circumscribe the targeted context. Thus, the following research questions guided this scoping review:

1. What educational strategies support the transformation and articulation of clinical data in health care students’ clinical reasoning?
2. What learning tasks are expected of students when exposed to these strategies?
3. What methods are used to assess students’ transformation and articulation of clinical data in clinical reasoning?

### Eligibility criteria

Eligibility criteria were defined according to the population–concept–context framework [16]. For the population, studies with students in health education programs across various academic levels (e.g., pregraduates and postgraduates) and disciplines were considered. Studies involving populations outside the health care field were considered if they also involved students in health education programs.

The central concept of this review was the semantic transformation and articulation of clinical data [7], implying a cognitivist understanding of the development of clinical reasoning skills [20]. Only articles that addressed these cognitive operations were considered. Additionally, clinical reasoning is a cognitive process underlying clinical judgment and decision-making [3, 21]. These terms, often used interchangeably [3, 22], were considered in the search strategy.

For the context, this review considered studies in academic and clinical settings across all geographic areas. We considered sources reporting educational strategies or assessment methods involving the transformation and articulation of clinical data in learning and teaching clinical reasoning. Educational strategies refer to the approaches, activities, and methods used to improve individual competencies [23]. Learning tasks or stimuli activate and control cognitive processes to facilitate successful learning [24]. Assessment methods, i.e., methods used to evaluate learning or progress within an educational strategy or as part of an educational program, involve OSCE or written or oral examinations [6]. Studies examining the development and applications of intelligent or clinical decision-support systems were excluded because technical or engineering procedures would deviate from the review’s objectives.

We considered primary studies with quasi experimental, experimental, observational, qualitative, and mixed-method designs in peer-reviewed journals in English or French. We also included gray literature, such as doctoral theses, dissertations, conference proceedings, and research reports. Exploring non-commercially published literature allowed us to access studies with null or negative results that might otherwise not be disseminated. Conference abstracts, protocols, editorials, expert opinions, commentaries, letters, book reviews, blogs and social media were excluded, considering that we were looking for scientific and educational literature with sufficient content to help answer our research questions. The search spanned from 1990 to the present to cover the period after Bordage introduced semantic qualifiers in medical education.

### Information sources and selection of evidence

A comprehensive three-step search strategy was implemented with the support of a health science librarian. Initially, an exploratory search was performed in the Cumulative Index of Nursing and Allied Health Literature (CINAHL Complete; EBSCOhost) and PubMed to identify keywords and Medical Subject Headings (MeSH) in articles on the review topic. In November 2022, we subsequently searched five databases: CINAHL (EBSCOhost), MEDLINE (Ovid), EMBASE (Ovid), PsycINFO (Ovid), and Web of Science (Clarivate). These databases were chosen because they allowed us to identify relevant health sciences and education articles. The search strategy was first developed in CINAHL and later adapted to other databases (see Appendix B). Since each database has its own indexing system, we identified the controlled vocabulary terms for each database separately to ensure the specificity and equivalence of the definitions of the chosen terms. Such sensitive research requires more time for filtering and selecting literature but lowers the risk of missing relevant literature that answered the research questions. We then employed a hand search approach to review the reference lists of the included records and searched for unpublished studies via the ProQuest Dissertations & Theses electronic database and Google Scholar.

After the initial database search was conducted, the retrieved references were imported into Covidence (Veritas Health Innovation, Melbourne, Australia) to facilitate screening and identify duplicate records. To ensure consistency and establish a shared understanding, three reviewers (TCM, MFD, and DA) independently assessed the eligibility of a randomly selected sample of 25 articles based on the predefined inclusion and exclusion criteria. Two meetings were conducted to refine the selection of the clinical reasoning criteria. Two of the three reviewers independently screened the titles, abstracts, and full texts according to the predefined selection criteria. In the event of any disagreements, a third reviewer was consulted.

### Data extraction and synthesis

Using structured data extraction in a Microsoft Word file, three reviewers (MFD, YB, BMU) independently extracted the following data: general information (i.e., authors, publication year, country, study design, study purpose and population), educational strategies to facilitate the articulation and transformation of clinical data (e.g., case studies, online modules, case-based activities), learning tasks solicited from learners (e.g., completing a postencounter form, writing notes, responding aloud to open-ended questions), assessment methods to analyze the articulation and transformation of clinical data (e.g., analysis approaches, tools, or rubrics), and relevant results. When the authors described tools or rubrics, we summarized the domains evaluated, criteria, and scoring system, if applicable. However, this scoping review did not aim to report or evaluate the psychometric properties of these assessment tools. After the initial extraction process, a third author reviewed the extraction forms for each article, resolving discrepancies if needed via a third extraction.

Study characteristics (e.g., publication year, location, population, and design) were synthesized using descriptive statistics. The extracted data were then examined for overall patterns to assist in synthesis. More precisely, they were analyzed using content analysis techniques inspired by Miles et al. [25]. The data analysis method involved three steps: (1) data condensation, (2) data display of similarities and differences, and (3) drawing and verifying conclusions. The results are presented via tables and a narrative text summarizing the main conclusions of the studies. We also created a contingency table showing the educational strategies and the learning tasks they triggered in the studies.

## Results

**Fig. 1:**
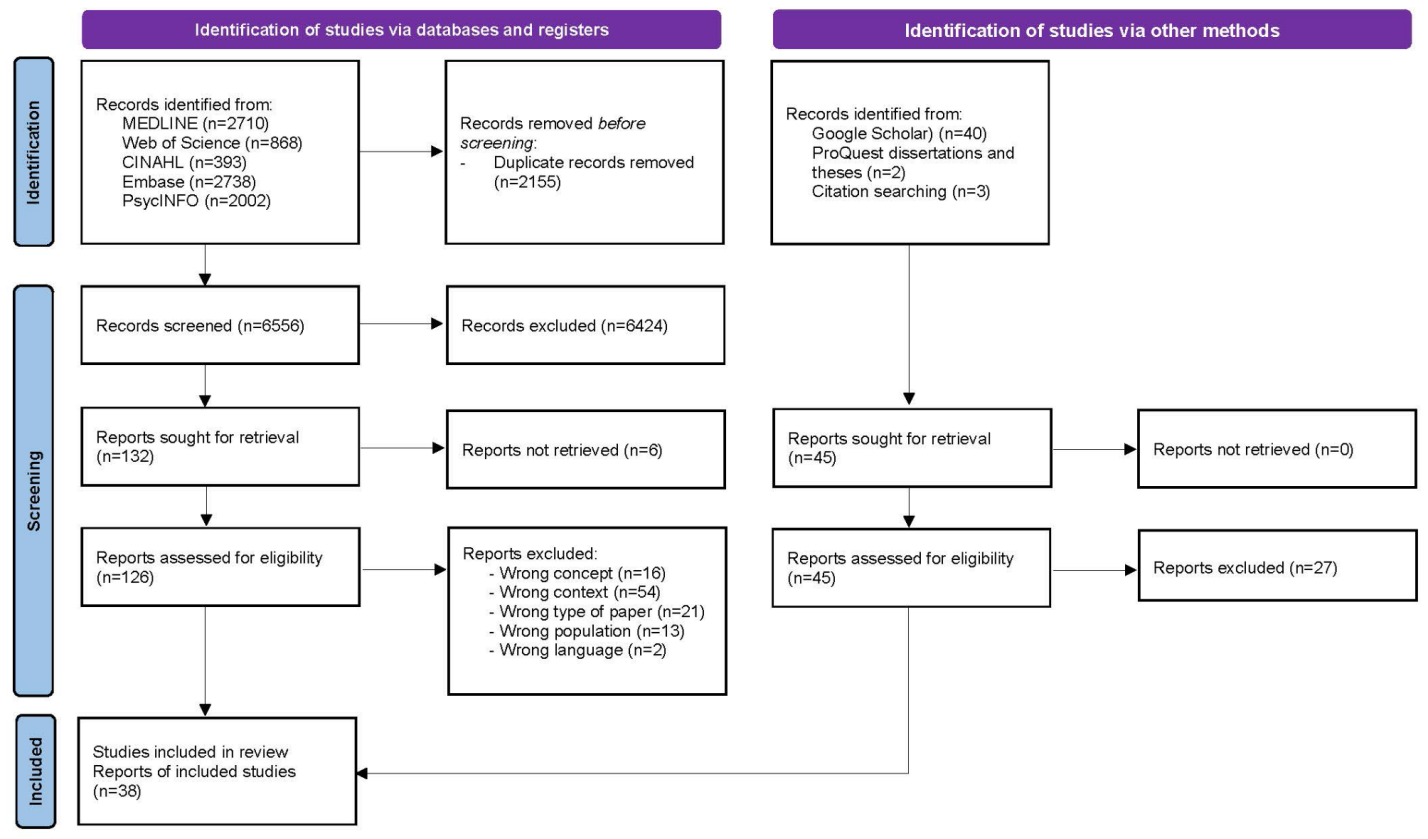
PRISMA-ScR flow diagram

From a pool of 6,656 unique records, 38 studies were included in this review (Fig. 1).

### Characteristics of the studies

Most studies were conducted in North America and Europe (*n =* 30, 79%) and focused primarily on medical students (*n* = 35, 92%). Most used observational designs (e.g., cohort, case‒control, and cross-sectional studies; *n* = 17, 45%), followed by methodological designs (*n* = 8, 21%) and quasiexperimental designs; *n* = 8, 21%). Experimental designs, including randomized controlled trials (RCTs), were used in five studies (13%). Studies aimed to (1) document how clinical reasoning is manifested in concrete observations of students and describe levels of clinical reasoning expertise (*n* = 12; 32%), (2) develop assessment tools or rubrics and explore their usability or psychometric qualities (*n* = 12; 32%), and (3) document the effects of various educational strategies on developing clinical reasoning (*n* = 12; 32%). Others explored the contextual elements impacting clinical reasoning (*n* = 2, 5%). Appendix C documents the characteristics of the studies, the educational strategies experimented, and the learning tasks expected of students when exposed to these strategies, the assessment modalities used as well as the results of the educational strategies on the development of clinical reasoning.

Early studies (i.e., the 1990s and 2000s) used predominantly descriptive designs documenting the feasibility or usability of using written notes or the think-aloud method to describe the quality of clinical reasoning development and differences in performance (e.g., diagnostic accuracy). The think-aloud method appears to provide more tangible access to students’ data transformation and articulation of clinical data compared to the written notes, particularly in conditions where students do not provide sufficient information in their notes to make enlightened judgments. Then, studies from the 2010s and beyond have used more quasi-experimental or experimental designs, and included students mostly from a single educational institution, which limits the generalizability of the results regarding the effects of educational strategies on the development of clinical reasoning and the influences of confounding variables related to the study context.

### Educational strategies to promote the transformation and articulation of clinical data in the context of clinical reasoning and related learning tasks

The educational strategies used to promote the transformation and articulation of clinical data in clinical reasoning for health care students included resolving written or computerized case-based scenarios (*n* = 13; 52%), simulated or real patient encounters, including standardized and virtual patients (*n* = 6; 24%), OSCE/or oral examinations (*n* = 3; 12%), concept mapping combined with virtual patients (*n* = 2; 8%) and problem-based learning (PBL) (*n* = 1; 4%). The learning tasks included collecting data, identifying key findings, grouping diagnoses with related key concepts, translating clinical information into semantic qualifiers, synthesizing the case aloud, responding aloud to prompt questions, writing a summary statement or a problem formulation (i.e., stating the diagnosis), writing a chart note or completing a postencounter form and answering written questions about problem formulation. Table 1 shows the educational strategies used in the studies alongside the learning tasks they triggered.

**Table 1:** Educational strategies in conjunction with learning tasks

Educational strategies						
		**Resolution of written or computerized cases-based scenarios**	**Simulated or real patient encounters**,** including SP and VP**	**OSCE or Oral examination**	**PBL**	**Concept mapping with VP**
Learning tasks	Collect data/identify key findings	Bonifacino et al. [26], Guerrasio and Aagaard [27] and Schaye et al. [28]	Hege et al. [29, 30], Heist et al. [31], Nendaz and Bordage [32] and Schaye et al. [28]	Diogo et al. [33] and Longo et al. [34]	Da Silva and Dennick [35]	
	Group the list of diagnoses with related key concepts	Choi et al. [36], Coderre et al. [37], Dore et al. [38], Elieson and Papa [39] and Guerrasio and Aagaard [27]	Hege et al. [29, 30], Nendaz and Bordage [32] and Schaye et al. [28]		Da Silva and Dennick [35]	Hege et al. [29, 30]
	Translate clinical information into semantic qualifiers	Bonifacino et al. [26], Bordage and Lemieux [40], Choi et al. [41] and Guerrasio and Aagaard [27]	Hege et al. [29, 30] and Wolpaw et al. [42]	Smith et al. [43]		Hege et al. [29, 30]
	Synthesize/present the case aloud	Bordage and Lemieux [40], Choi et al. [36, 41], Guerrasio and Aagaard [27], Mlika et al. [44] and Schaye et al. [28]	Chang et al. [45], Nendaz and Bordage [32], Schaye et al. [28] and Wolpaw et al. [42]	Longo et al. [34] and Smith et al. [43]	Da Silva and Dennick [35]	
	Respond to prompt questions aloud	Bordage and Lemieux [40] and Guerrasio and Aagaard [27]	Nendaz and Bordage [32]	Diogo et al. [33], Longo et al. [34] and Smith et al. [43]		
	Write a summary statement or a problem formulation/state the diagnosis	Auclair [46], Bonifacino et al. [26], Choi et al. [36, 41], Dore et al. [38], Elieson and Papa [39], Eva et al. [47], McQuade et al. [48], Patel et al. [49] and Schaye et al. [28]	Hege et al. [29, 30], Heist et al. [31] and Nendaz and Bordage [32]		Da Silva and Dennick [35]	
	Write a chart note or complete a PEF		Baker et al. [50] and Nendaz and Bordage [32]	Diogo et al. [33]		
	Respond to written questions	Choi et al. [36], Coderre et al. [37], Eva et al. [47], Guerrasio and Aagaard [27], McQuade et al. [48] and Patel et al. [49]	Baker et al. [50] and Nendaz and Bordage [32]			

**OSCE**, Objective Structured Clinical Examination; **PBL**, Problem-based learning; **PEF**, postencounter form **SP**, standardized patient; **VP**, virtual patient

The most frequently documented strategies were resolving written or computerized case-based scenarios and simulated or real patient encounters, including standardized and virtual patients. For example, Nendaz and Bordage [32] measured the effect of an educational strategy on diagnostic argumentation and diagnostic accuracy. Workshops incorporating videotapes of students’ examinations of standardized patients were used with the intervention group incorporating various learning tasks. The results showed that the intervention group used significantly more semantic qualifiers than the control group (*p =* 0.006). However, no differences were found in the number of semantic qualifiers used in write-ups or in diagnostic accuracy (*p >* 0.56). Researchers hypothesized that semantic qualifiers may be necessary for efficient problem representation but may be insufficient for developing diagnostic reasoning competency [32]. Conversely, in a randomized controlled trial conducted by Wolpaw et al. [44], students completing a family medicine clerkship were assigned to either: 1- the SNAPPS^1^ method, a structured case presentations technique; 2- a standardized educational strategy of deliberate feedback from the clinical supervisor, or 3- a traditional educational strategy. Results showed that students in the SNAPPS intervention group had more concise presentations (*p* < 0.000), proposed more differential diagnoses and relevant justifications and sought new information more frequently to guide their clinical reasoning than the other groups.

In the study of Choi et al. (2020), the effectiveness of combining practice with reflection and immediate feedback in traditional dermatology electives was investigated to improve resolution of complex situations involving evaluation of skin lesions. Medical students (*n =* 87) enrolled in a 2-week dermatology elective course participated in the study. The intervention group was engaged in a 2-h training course comprising resolution of written clinical cases, the lecture group completed a 1-hour lecture followed by 1-hour outpatient clinic and the non-intervention group completed 2-hours of outpatient clinic. Assessment tests were administered before and after a 2-week course. Students had to write down the two most likely diagnostics in written clinical cases with photographs. The results showed that the mean score was higher in the experimental group (7.5 ± 1.3) than in the lecture (5.7 ± 1.6) and no intervention (5.6 ± 1.3) groups (*p* < 0.001).

Additionally, Heist et al. [31] explored first-year resident physicians’ styles of summarizing cases in a summary statement and evaluated the effectiveness of virtual simulations in improving the quality of these summaries. Resident physicians were assigned to randomized sequences of five virtual modules rolled out at six-day intervals. During each module, participants created a free-text summary of a case and then accessed an expert model of a summary statement. From modules 1 to 5, increases were observed in the use of a narrative summary statement style (*p =* 0.016), the summary statement clinical reasoning quality score (*p =* 0.021), and the percentage of semantically driven summary statements (*p =* 0.003).

Clinical reasoning curricula, including lectures, assignments, case-based discussions and a series of written examinations, are implemented in health education programs to solicit a variety of learning tasks, promote the development of students’ clinical reasoning, and provide tailored remedial teaching plans [26–28, 36].

In Bonifacino et al. [26]’s study, aiming to evaluate, among other things, the impact of a clinical reasoning curriculum on students’ knowledge and skills, workshops and online modules were used in the intervention group. The control group completed the modules outside the study period. The results show that the intervention group demonstrated superior performance in the knowledge quiz (67% vs. the control group’s 54%, *p <* 0.001) and superior written reasoning skills in the data synthesis (2.3/3 vs. the control group’s 2.0/3, *p =* 0.02) and in the diagnostic reasoning (2.2/3 vs. the control group’s 1.9/3, *p =* 0.02) portion of the medical admission notes. The study of Schaye et al. [28] also documented the positive contribution of a clinical reasoning curriculum. The researchers tested whether a clinical reasoning curriculum, embedded within the workplace, would improve the diagnostic reasoning process, and the application of core concepts taught in the curriculum. First-year medical residents (*n =* 71) were divided into 3 groups (Group 1: no intervention; Group 2: Part 1 = Iterative resolution of written clinical cases; Group 3: [Parts 1 & 2] Part 2 = embedded within an inpatient clinical rotation and meeting with a senior). Significant differences were found in the application of clinical reasoning concepts [no intervention 1.6/3 (0.65) compared to partial 2.3/3 (0.81) and full 2.2/3 (0.91), *p =* 0.05] as well as in describing cases in problem representation format [no intervention 1.2/3 (0.38) and partial 1.5/3 (0.55) compared to full 2.1/3 (0.93), *p =* 0.004].

What is common in these curricula are the worked examples that model the clinical reasoning process of experienced health care professionals, particularly the cognitive process of transforming and articulating clinical data [26–28, 36].

### Methods and tools used to assess individuals’ transformation and articulation of clinical data

The use of key concepts and professional vocabulary, including semantic qualifiers, was evaluated in twenty-one studies (55%) through learners’ written notes, post encounter forms, summary statements, or written summaries/diagnostic justification essays of case-based or real clinical situations [26, 27, 29–33, 36, 46, 48, 50–60]. Some researchers used Bordage et al. [11]’s simplified method (i.e., discourse categorized as reduced, scattered, elaborated or compiled) to assess students’ written notes [34, 50]. Moreover, Choi et al. [41] used written examinations to determine whether summary statements were complete and concise and demonstrated the use of semantic qualifiers. Other researchers have used specific rubrics to assess the quality of student discourse, including criteria of completeness, conciseness, and appropriate use of semantic qualifiers, which are often based on a predetermined list of expected semantic qualifiers [29, 30, 32, 33, 48, 51]. For example, Heist et al. [31] used an institutional scoring rubric for the summary statement for each virtual simulation. Assessors evaluate semantic qualifiers by identifying whether each summary statement is semantically driven.

Many researchers have emphasized the importance of using key concepts and professional vocabulary, including semantic qualifiers, with the think-aloud approach as an assessment method (*n* = 13; 34%) [20, 27, 32–35, 40, 42–45, 61, 62]. In the study of Diogo et al. [33], verbalizations were analyzed as ‘strong’ encapsulations encompassing several ‘smaller’ clinical concepts (e.g., heart failure includes dyspnea, orthopnea, and peripheral edema), whereas ‘weak’ encapsulations represented only simple word transformations into medical terminology. In contrast, in the study by Da Silva and Dennick [35], clinical reasoning was assessed by measuring the frequency of words associated with the grammatical category of ‘subordinating conjunctions’ (i.e., ‘if,’ ‘then,’ ‘when,’ and ‘because’), which are used to join a subordinate (dependent) clause to a main (independent) clause. Differences in semantic and grammatical filters between PBL sessions were observed and related to the focus of the sessions (e.g., investigating causality, disease probability, relationships between data). In the study by Durning et al. [61], qualitative utterances from think-aloud activities were converted into numerical measures of cognitive load to explore the influence of contextual factors on clinical reasoning performance.

Finally, researchers have used various variables, such as presentation length, conciseness, and completeness, to evaluate the quality of discourse and clinical reasoning [42, 44]. Furthermore, the coherent and structured organization of knowledge, which integrates key concepts and semantic qualifiers, has been assessed via concept mapping [29, 30, 34, 43]. Written exams (e.g., problem-solving questions, extended-matching R-type questions, evolving multistep exams, or script concordance tests) were used to assess students’ level of knowledge and the quality of professional vocabulary used to describe clinical data [27, 37, 63].

Several researchers have used or developed tools to evaluate learners’ overall clinical reasoning process, including transforming and articulating clinical data. These tools include the Interpretive summary, Differential diagnosis, Explanation of reasoning, and Alternatives (IDEA) tool [26, 54] and its revised versions [57], Patient Note Scoring (PNS) [59], and other rubrics [31, 53, 55, 58, 62]. The synthesis of these methodological study’s data is presented in Appendix D, where the aims pursued by the researchers, the target population, the tools or rubrics developed or used as well as the educational program context, including the nature of the evaluation, are documented. Table 2 presents the description of the tools or rubrics, details on the domains evaluated, and the scoring system where applicable.

**Table 2:** Description of the tools or rubrics

Rubric or tool	Description
IDEA tool [54]	The Interpretive summary, Differential diagnosis, Explanation of reasoning, and Alternatives (IDEA) is a 15-item instrument that includes four elements: interpretive summary (I), differential diagnosis (D), explanation of reasoning (E), and alternatives (A). The tool uses a 3-point Likert scale.
The revised IDEA assessment tool [57]	The revised IDEA assessment tool includes the same four domains as the IDEA assessment tool but has more detailed descriptive prompts, new Likert scale anchors, and a score range of 0 to 10.
PBEAR [62]	The Problem Representation, Background Evidence, Analysis, Recommendation (PBEAR) modified from the Situation, Background, Assessment, Recommendation (SBAR) framework. The tool contains scoring choices ranging from 1 to 4 and dichotomous options (yes/no) to assess CR components. The tool uses a 5-point Likert scale with descriptive anchors.
CRT [64]	The Clinical Reasoning Task (CRT) contains a taxonomy of 24 tasks that physicians use to reason through clinical cases.
PNS rubric [59]	The Patient Note Scoring (PNS) covers the following three domains with items scored on a scale of 1–4 points: documentation of pertinent history and exam findings, differential diagnosis, and diagnostic workup.
SSAR rubric [56]	The summary statement assessment rubric (SSAR) is a 5-domain instrument to evaluate the CR documented within summary statements. The domains are factual accuracy, appropriate narrowing of the differential diagnosis, information transformation, semantic qualifier use, and a global rating.

Recently, Cianciolo et al. [52] explored the potential of machine learning technologies to score students’ diagnostic justification essays. The machine scoring system utilized several semantically based metrics from natural language processing to assess the quality of students’ competencies. The results showed that the machine scores correlated more strongly with faculty ratings than faculty ratings did with each other (machine: 0.28–0.53, faculty: 0.13–0.33) and were less case-specific.

## Discussion

This scoping review aimed to provide a comprehensive overview of the evidence on the transformation and articulation of clinical data in health care clinical reasoning education. Various educational strategies have been identified, including resolving written or computerized case-based scenarios; simulated or real patient encounters, including standardized and virtual patients; OSCE/or oral examination; and concept mapping combined with virtual patients and PBL. The identified learning tasks focused primarily on collecting data, identifying key findings, summarizing situations, and translating clinical information into semantic qualifiers. Furthermore, the review uncovered assessment methods and rubrics incorporating the transformation and articulation of clinical data for evaluating clinical reasoning. These findings highlight the diversity of educational strategies and assessment tools in the field. The results reveal consistency between educational strategies, learning tasks, and assessment methods to ensure constructive alignment in teaching and assessing clinical reasoning. Constructive alignment [65] in higher education implies coherence between learning objectives, educational strategies, and assessment tools. In health education programs, educators also consider how health care professionals act and reason in their clinical environment to make learning tasks authentic and meaningful [66, 67].

Analyzing students’ discourse provides insights into the richness and organization of their clinical knowledge, aligning with the theoretical framework underlying this review [8–10, 19, 40]. A fundamental and well-structured knowledge base is essential for sound clinical reasoning and appropriately utilizes related cognitive operations. The primary learning tasks retrieved in studies (i.e., collecting data/identifying key findings, grouping diagnoses with associated key concepts, and translating clinical information into semantic qualifiers) refer to problem representation, a cognitive process of abstraction illustrating an interpretive understanding of a problematic situation [4, 10, 32, 48]. To develop an appropriate problem representation, students must recognize key features of a situation based on their professional knowledge and clinical experiences with similar situations. In short, these findings support a cognitivist perspective on learning, teaching and assessing clinical reasoning, where acquiring and organizing professional knowledge is crucial [20].

The results show that oral or written information exchanges (i.e., documentation) leave traces or evidence that can be used to deduce the quality of students’ clinical reasoning process. While written notes are a ubiquitous part of student education, they are still underutilized in the formal assessment of clinical reasoning despite their high content validity and alignment with clinical documentation practices [6]. Analytic scoring rubrics are mainly used to evaluate oral or written notes. Generally, rubrics for analyzing student discourse include criteria for factual accuracy, conciseness and completeness, appropriate narrowing of differential diagnosis, transformation of information, and use of semantic qualifiers [31, 53, 55, 58, 62]. Some rubrics also integrate significant elements related to the patient (in clinical settings or simulated situations) and the practice environment, suggesting a more situated or ecological theoretical perspective of clinical reasoning [20]. Using one criterion as a global assessment within a rubric [51, 54–57] or using a holistic rubric [52] can more accurately capture the communicative and contextual aspects of clinical reasoning [6].

Although the objectives of the scoping review were not to document the effect of educational strategies on student performance in clinical reasoning, the narrative synthesis of the data shows favorable results in studies integrating various educational strategies in a clinical reasoning curriculum [26–28, 36] compared to an educational strategy used alone or episodically [32, 42, 44]. This confirms that clinical reasoning is a complex cognitive construct [2]. It also highlights the importance of using a robust evaluation model as a framework to systematically guide the design, implementation and evaluation of educational strategies and provide judgment on their benefits and impacts to develop clinical reasoning, especially those focused on transforming and articulating clinical data.

### Implications for research and educational practice

A first recommendation for research would be to consider future directions of research on interdisciplinary clinical reasoning. The results from this review strongly represent the medical discipline, which is not surprising considering the nature and essence of medical practice involving diagnosing, differentiating diagnoses, and establishing treatment plans. However, with the current transdisciplinary practice and advanced professional roles (e.g., specialized nurse practitioner), there is a need for more empirical exploration of cognitive operations related to clinical reasoning in other health disciplines (e.g., physiotherapy, occupational therapy, speech-language pathology, nursing). The theoretical framework used in this review may contain blind spots on disciplinary perspectives about clinical reasoning by other health professionals. For example, one might ask how clinical reasoning in nursing or occupational therapy differ from that in medicine, and what are the unique challenges of learning and teaching clinical reasoning in these fields? As clinical reasoning in these different disciplines may differ or overlap, it might also be interesting to study how interdisciplinary teams approach clinical reasoning, especially for shared or distributed decision-making.

A second recommendation for research would be to consider future directions on AI tools interfering/supporting the learning and teaching of clinical reasoning. AI tools can model and reproduce human language capabilities and have the potential to make simulated case studies more dynamic than traditional educational strategies, by enabling, for example, authentic patient-health professional interactions or conservations [68], to trigger students’ transformation and articulation of clinical data. Educators are then encouraged to make relevant use of AI in teaching clinical reasoning [13], but challenges remain in their implementation. For example, Cianciolo et al. [52] investigated the potential of machine learning technologies to score students’ diagnostic justification essays. This is a significant advantage of AI in providing automated feedback to students, which could be standardized and reduce the time investment of educators. However, the acceptability and usefulness of these technologies for assessing students’ clinical reasoning still raise some questions and warrant further research. Furthermore, the use of AI creates ethical issues such as the importance given to algorithmic decision-making linked to clinical reasoning rather than the human approach (e.g., empathy, sensitivity or gut feeling to detect changes in patients’ conditions and human conservations with patients), while the latter is an essential element and interwoven with other components of the cognitive process.

While analyzing students’ discourse can provide didactic value for promoting learning or teaching clinical reasoning and its assessment, the methods to achieve this are less clear. However, the results of this scoping review offer relevant avenues for educators. The main recommendation in terms of educational practices would be to consider various educational strategies to promote the development of students’ clinical reasoning. Most of the modalities listed include articulating students’ knowledge through case presentations, discussions and reflection, writing medical notes, and completing postencounter forms. On these occasions, educators could prompt students to create one-sentence summary statements of the case via semantic qualifiers. When using written or computerized case-based situations, instructions such as ‘State the diagnosis you are considering’; ‘List the facts for or against each possibility’; ‘State your conclusion and the main reason for this conclusion’, etc. support the student’s written reasoning process [29, 49, 53, 55, 63]. By promoting the use of key features and encouraging the construction of accurate problem representations, students are expected to develop a deeper understanding of clinical information, facilitating better communication and reasoning in clinical settings.

Educators could also articulate their own representation of the problem aloud or in writing, illustrating their interpretation of the situation, the types of semantic qualifiers used, and the discriminating features they sought to refine the clinical reasoning process in such situations (e.g., Here’s how I think the situation might be resume .). In other words, providing students with worked examples can help prevent cognitive overload, allowing them to process and articulate clinical data better. This can be achieved using standardized patient encounter videos [32, 42], case-based situations [32, 41] or real situations in clinical settings where the educator presents aloud their interpretation of the situation following a patient encounter [28]. Examples illustrating different categories of discourse, as in Appendix A, could be used with students to demonstrate how the transformation and articulation of clinical data are illustrated through information exchanges between health professionals. This learning can also be supported using communication structuring tools (e.g., SNAPPS method, the Problem Representation, Background Evidence, Analysis, Recommendation [PBEAR] tool) [42, 44, 62].

Students should be encouraged to compare and contrast probable diagnoses in simulated or actual practice situations. Asking students to prioritize and explain a restricted list of diagnostic possibilities could help them develop links between the data and contrast them with typical presentations while remaining attentive to contextual data or factors. Many studies have used pedagogical questioning in students’ learning tasks. For example, while using OSCEs or encounters with real or standardized patients, interviews allow prompting the learners to verbalize the cases aloud [33, 34, 45, 50]. Probing questions such as, ‘What diagnosis did you reach?’ ‘What led you to this diagnosis?’ ‘Do you need any additional information to reach a final diagnosis?’ ‘If this finding (e.g., ‘gradual onset’ is discovered), how does it change your thoughts?’ could be used to document learners’ clinical reasoning processes [33, 34, 45, 50]. These questions targeted higher taxonomic levels, including clinical data evaluation and comparison, clinical data analysis and transformation into professional vocabulary, and synthesis. This underscores the importance of pedagogical competencies in addition to the professional knowledge of educators in clinical and academic settings.

### Limitations

Despite its robust design, this scoping review has several limitations. The review primarily included peer-reviewed journal articles and may have resulted in the exclusion of some relevant studies. As such, some relevant gray literature may be excluded from our findings. Excluded studies on developing and applying intelligent or clinical decision support systems could contain additional information to answer the review questions. Indeed, the broad keywords used in the search strategy may not capture all specialized studies. So, there may be potential biases in the methodological choices impacting the result we obtained.

Moreover, considering only publications in English and French could exclude relevant papers written in other languages. The exclusion of non-English and non-French studies may have affected the scope and possible insights from non-Western perspectives that could have enhanced the global relevance of the manuscript. Also, most studies retrieved (more than 90%) were conducted with medical students, thus limiting the generalizability of the results to other disciplines. As mentioned in the discussion section, theoretical or disciplinary frameworks can potentially be used in other health disciplines to understand or study the development of clinical reasoning, even if the cognitive processes remain relatively similar. Broadening knowledge from common and differentiated disciplinary perspectives (e.g., nursing, physiotherapy, occupational therapy, speech-language pathology) could thus further nuance the results we obtained in this review.

Finally, the review’s currency was affected by the time required to analyze all the references uncovered in our search. Thus, some new developments in the semantic transformation and articulation of clinical data during clinical reasoning education since November 2022 may not have been included, although the principal investigator regularly received alerts from databases. These alerts informed new studies on the initial research strategy and were considered during the ongoing data analysis process.

## Conclusions

By synthesizing evidence on the semantic transformation and articulation of clinical data during clinical reasoning education, this review aims to refine strategies and assessment methods used in academic and continuing education programs. The insights gained from this review will help educators develop more effective approaches for teaching/learning clinical reasoning. Additionally, the results may offer solutions for addressing challenges related to assessing clinical reasoning and ensuring that assessment tasks accurately reflect learners’ developing competencies and educational progress. Further research is needed to deepen our understanding of teaching, learning, and assessing clinical reasoning as a complex competency and phenomenon, particularly in the current context where AI increasingly plays a role in collective cognition.

## Electronic supplementary material

Below is the link to the electronic supplementary material.

Supplementary Material 1

Supplementary Material 2

Supplementary Material 3

Supplementary Material 4

Supplementary Material 5

## Data Availability

The datasets generated and/or analyzed during this study are available from the corresponding author upon reasonable request.

## Footnotes

1 SNAPPS = Summarize, Narrow, Analyze, Probe, Plan, and Self-study.

## Abbreviations

AI: Artificial intelligence
CR: Clinical reasoning
OSCE: Objective structured clinical examination
PBL: Problem-based learning
PRISMA-ScR: Preferred reporting items for systematic reviews and meta-analyses extension for scoping reviews
PRISMA-P: Preferred reporting items for systematic review and meta-analysis protocols
