# Supplementary material for: Transformation and articulation of clinical data to understand students’ clinical reasoning: a scoping review
Source: BMC Med Educ. 2025 Jan 12;25:52. doi: 10.1186/s12909-025-06644-7 (PMC11725190; doi:10.1186/s12909-025-06644-7)
Supplement: Supplementary file 1 — Supplementary Material 1 [file 12909_2025_6644_MOESM1_ESM.docx]

**Appendix A. Description of category of discourse with examples of nurse-to-physician exchange of information**

Mr. George, 55 year-old man, was admitted yesterday on the intensive care unit for unstable angina with a history of dyslipidemia and sleep apnea.. Mr. George is complaining of nausea and an sudden chest pain. He describes the pain as chest tightness at 9/10, with radiation to the left arm and jaw. His respirations are 30 breaths/min with desaturation to 86% on room air. ST segment elevation is currently observed in leads V2, V3 and V4 on the electrocardiogram.. Mr. George’ heart rate is 120 and his blood pressure is 85/55. Mr. George is alert but agitated.

| **Category of discourse^[[1]](#footnote-2)^** | **Description** | **Examples of nurse-to-physician exchange of information** |
| --- | --- | --- |
| Reduced | Discourse that lacks any effort of semantic transformation, with no connections between patient data and knowledge | “Dr. X, this Megan Smith, RN, from the intensive care unit at X Hospital. I’m calling about your patient, Mr. George, 55 year-old. He is complaining of nausea and chest pain. I don't know what's going on, but Mr George doesn't feel well” |
| Scattered | Discourse that exhibits limited semantic transformation and disordered hypotheses, which do not reference the obtained data and are listed without contrasts | “Dr. X, this is Megan Smith, RN, from the intensive care unit at X Hospital. I’m calling about your patient, Mr. George, 55 year-old. Mr. George is complaining of nausea and he is suffering. His vital signs are : blood pressure at 85/55 and the heart rate is 120 bpm. He presents 30 breaths/min with a saturation at 86% on room air. Something is happening, Mr George is not going well. Is it a digestive, respiratory or a heart disorder? I don’t know. Do we need to do a blood sugar test?” |
| Elaborated | Discourse with numerous semantic transformations used judiciously to contrast hypotheses | “Dr. X, this is Megan Smith, RN, from the intensive care unit at X Hospital. I’m calling about your patient, Mr. George, a middle-age man, is complaining of nausea and an sudden chest pain. He was admitted yesterday for unstable angina with a history of dyslipidemia and sleep apnea. He describes the pain as chest tightness at 8/10, with radiation to the left arm and jaw. Mr. George is tachypneic at 30 breaths/min with desaturation to 86% on room air. ST segment elevation is currently observed in leads V2, V3 and V4 on the electrocardiogram, illustrating cardiac distress. Mr. George is hypotensive with a blood pressure of 85/55 and he has sinus tachycardia at 120 bpm. Mr. George is alert but agitated due to the severe pain. I started oxygen therapy and ensured that an IV line was accessible. I recommend an immediate evaluation. Do you agree?” |
| Compiled | The individual immediately recognizes semantic datasets associated with a clinical hypothesis | “Dr. X, this is Megan Smith, RN, from the intensive care unit at X Hospital. I’m calling about your patient, Mr. George. Mr. George, a middle-age man, appears to be currently experiencing an acute cardiac distress, observed by a ST segment elevation in leads V2, V3 and V4 on the electrocardiogram. He also presents signs of shock, i.e, hypotension at 85/55 and sinus tachycardia at 120 bpm, but he is alert. Mr George is agitated, due to the severe and sudden retrosternal pain at 9/10, with radiation to the left arm and jaw. Mr. George is tachypneic with desaturation to 86% on room air. He was admitted yesterday for unstable angina with a history of dyslipidemia and sleep apnea. I started oxygen therapy and ensured that an IV line was accessible. I recommend an immediate evaluation. Do you agree?” |

1. Note: Adaptation of Bordage et al. [11] [↑](#footnote-ref-2)
