# Supplementary material for: Transformation and articulation of clinical data to understand students’ clinical reasoning: a scoping review
Source: BMC Med Educ. 2025 Jan 12;25:52. doi: 10.1186/s12909-025-06644-7 (PMC11725190; doi:10.1186/s12909-025-06644-7)
Supplement: Supplementary file 2 — Supplementary Material 2 [file 12909_2025_6644_MOESM2_ESM.docx]

**Appendix B. Search strategy**

| No. | Database | Search | Query | Records  retrieved |
| --- | --- | --- | --- | --- |
| 1 | CINAHL | #1 | (MH “Diagnostic Reasoning”) OR (MH “Decision-making, Clinical+”) | 1580 |
|  |  | #2 | (Clinical N2 (Reason* OR judgement* OR think* OR Prediction*)) OR (Diagnos* N2 (Reasoning OR Differential)) OR (Decision* N2 clinical) OR (Medical N2 reasoning) | 157334 |
|  |  | #3 | (S1 OR S2) | 157334 |
|  |  | #4 | (MH “Semantics”) OR (MH “Psycholinguistics”) | 9209 |
|  |  | #5 | Semantic* OR psycholinguistic* | 16357 |
|  |  | #6 | (S4 OR S5) | 16357 |
|  |  | #7 | (S3 AND S6) | 402 |
|  |  | #8 | S7 AND LA (english OR french) AND DT 19900101-20231231 | 393 |
| 2 | MEDLINE | #1 | diagnosis/ or clinical decision-making/ or clinical reasoning/ or diagnosis, differential/judgment/ or problem solving/ | 546343 |
|  |  | #2 | (Clinical adj2 (Reason* or judgement* or think* or Prediction*)) ab,kf,ti. | 18126 |
|  |  | #3 | (S1 OR S2) | 686049 |
|  |  | #4 | Semantics | 28442 |
|  |  | #5 | "Semantic*ab,kf,ti. | 45328 |
|  |  | #6 | (S4 OR S5) | 57463 |
|  |  | #7 | (S3 AND S6) | 2964 |
|  |  | #8 | limit S7 to ((english or french) and yr="1990 -Current") | 2710 |
| 3 | EMBASE | #1 | clinical reasoning/ or diagnostic reasoning/diagnostic accuracy/ or diagnostic reasoning/ or differential diagnosis/ or dual diagnosis/decision making/ or clinical decision making/ or medical decision making/ | 1085848 |
|  |  | #2 | (Clinical ADJ2 (Reason* OR judgement* OR think* OR Prediction*)) OR (Diagnos* ADJ2 (Reasoning OR Differential)) OR (Decision* ADJ2 clinical) OR (Medical ADJ2 reasoning) | 593642 |
|  |  | #3 | (S1 OR S2) | 1225918 |
|  |  | #4 | Semantic/ | 30187 |
|  |  | #5 | Semantic* OR semantic qualifiers.ab,kf,ti. | 6653 |
|  |  | #6 | (S4 OR S5) | 32098 |
|  |  | #7 | (S3 AND S6) | 2921 |
|  |  | #8 | S7 AND limit to ((english or french) and yr="1990 -Current") | 2738 |
| 4 | APA PsyINFO | #1 | decision making/ or judgment/ or problem solving/Differential Diagnosis/ or Medical Diagnosis/ or Diagnosis/ or Dual Diagnosis/ | 210297 |
|  |  | #2 | (Clinical ADJ2 (Reason* OR judgement* OR think* OR Prediction*)) OR (Diagnos* ADJ2 (Reasoning OR Differential)) OR (Decision* ADJ2 clinical) OR (Medical ADJ2 reasoning) | 40647 |
|  |  | #3 | (S1 OR S2) | 234315 |
|  |  | #4 | Semantic | 20942 |
|  |  | #5 | "Semantic*"ab,id,ti | 55327 |
|  |  | #6 | (S4 OR S5) | 56246 |
|  |  | #7 | (S3 AND S6) | 2431 |
|  |  | #8 | S7 AND LANGUAGE: (English OR French) and yr= “1990-Current”) | 2002 |
| 5 | Web of Science | #1 | (Clinical NEAR/2 (Reason* OR judgement* OR think* OR Prediction*)) OR (Diagnos* NEAR/2 (Reasoning OR Differential)) OR (Decision* NEAR/2 clinical) OR (Medical NEAR/2 reasoning) | 200949 |
|  |  | #2 | Semantic* OR psycholinguistic* | 269676 |
|  |  | #3 | (S1 OR S2) | 910 |
|  |  | #4 | S3 AND (PY=(1990-2023)) AND LANGUAGE: (English OR French) | 868 |
|  | TOTAL |  |  | 8711 |
