# Supplementary material for: Transformation and articulation of clinical data to understand students’ clinical reasoning: a scoping review
Source: BMC Med Educ. 2025 Jan 12;25:52. doi: 10.1186/s12909-025-06644-7 (PMC11725190; doi:10.1186/s12909-025-06644-7)
Supplement: Supplementary file 3 — Supplementary Material 3 [file 12909_2025_6644_MOESM3_ESM.docx]

**Appendix C. Characteristics of studies**

| Authorship  country | Study type | Aims | Population | Educational strategies /Learning tasks | Assessment methods/ Tools or rubric used | Results |
| --- | --- | --- | --- | --- | --- | --- |
| Auclair (2007)  Canada | Observational | To describe how students formulate the problem of a complex case | Third-year medical students (*n* = 32) | **Resolution of fictitious written clinical cases**   - Synthesize the case, write the problem formulation, and state the diagnosis | Analysis of the presence/absence of the essential elements, the use of higher-order concepts, and the mention of the relations between them. | Diagnostic accuracy was significantly associated with the use of higher-order concepts and relations between concepts. When presented with an already formulated problem, 19/25 students made the correct diagnosis (*p* < 0.05). |
| Baker et al. (1999)  United  States | Observational | To investigate the written record as a source of information about how the discourse is organized | Residents in family medicine (*n* =8) and internal medicine (*n* = 9) | **Encounter with SP and real patients**   - Write SOAP notes and answer questions about the formulation of diagnoses | Discourses categorized using **Bordage’s simplified method** [3]. Written notes were analyzed according to the degree to which SQs had been used to actively contrast diagnoses. | The residents provided sufficient information in their SOAP notes to make enlightened judgments in only 6% of the cases. A simple listing of diagnoses is insufficient for proper semantic classification. |
| Bonifacino et al. (2019)  United States | Quasi-experimental | To evaluate the impact of a CR curriculum on students’ knowledge of CR concepts and skills and their perception of CR learning | Third-year medical students completing an internal medicine clerkship (*n* = 67) | Intervention group: **Workshops and online modules of fictitious clinical cases**  Control group: Complete modules outside the study period   - Identify key findings, translate clinical information into SQ; write a SS, and state a diagnosis. | Completion of a 20-point CR concept quiz.  The **IDEA tool** was used after the completion of the curriculum. | The intervention group demonstrated superior performance in the knowledge quiz (67% vs. the control group’s 54%, *p* < 0.001) and superior written reasoning skills in the data synthesis (2.3 vs. the control group’s 2.0, *p* = 0.02) and in the diagnostic reasoning (2.2 vs. the control group’s 1.9, *p* = 0.02) portion of the admission notes |
| Bordage and Lemieux (1991)  Canada | Observational | To use structural semantics to interpret clinical data and generate diagnoses | Medical students (*n* = 29) and specialists (*n* = 10) | **Resolution of fictitious written clinical cases** (neurology, *n* = 3; gastroenterology, *n* = 4).   - TA | **Structural semantic analysis** used**.** Participants’ discourse was divided into five types of organization: (1) full economy, (2) full dispersion (3) empty dispersion, (4) empty economy, and (5) intermediate. | A positive relationship was found between the number of semantic axes used and the accuracy of the diagnoses (.90 for neurology cases (*p* < 0.00; .067 for gastroenterology cases, *p* <.0.00). |
| Chang et al. (1998)  United States | Case study | To explore diagnosticians’ success during case presentations | Third-year medical students (*n* = 19) and expert rheumatologists (*n* = 4) | **Examinations of standardized patients**   - Present the case | Each presentation was divided into arguments representing a distinct line of reasoning and instances when discrete data from the clinical presentation were transformed into SQs. | The successful diagnosticians showed more thorough and relevant problem representations and did more simultaneous comparison and contrast of diagnoses (*p* <0.01) |
| Choi et al. (2020)  South Korea | Randomized controlled trial | To investigate the effectiveness of combining practice with reflection and feedback in traditional dermatology electives to improve evaluation of skin lesions | Fourth-year medical students enrolled in a 2-week dermatology elective course (*n* = 87) | Intervention group: 2-h **training comprising resolution of 10 fictitious written clinical cases**   - Write a description of using SQs. - Verbalize aloud the observations using SQ   Lecture group (1 h lecture followed by 1 h of outpatient clinic)  No-intervention group (2 h of outpatient clinic) | Testing was administered before and after a 2-week course. Students wrote down the two most likely diagnostics of 20 written clinical cases with photographs. They were rewarded with one point for each correct diagnosis. | After completing the 2-week rotation, for the training set, the mean score was higher in the experimental group (7.5 ± 1.3) than in the lecture (5.7 ± 1.6) and no intervention (5.6 ± 1.3) groups. |
| Choi et al. (2023)  United States | Quasi-experimental | To evaluate the value of explicitly teaching CR theory and cognitive bias | First-year medical students (*n* = 106) | Curriculum including **lectures, assignments, case-based discussions, and written examinations**   - Write a problem list and a problem representation. - Address alternative hypotheses and additional information needed, etc. | Written CR examinations were used to determine whether the problem representation was considered (1) complete and (2) concise and (3) whether it demonstrated the use of SQs. | 254 students (80%) showed complete problem representation, and 199 responses (63%) were considered concise. SQs  were included in 195 (92%) problem representations  analyzed in the first clinical reasoning examinations. |
| Coderre et al. (2009)  Canada | Cross-sectional observational | To study the knowledge structure and examine its association with diagnostic performance | Medical students (*n* = 91) | **Concept-sorting tasks of 4 problems**:   - Regroup list of diagnoses with related key concepts | Knowledge structures were categorized as either problem-specific or generic. For the problem-solving questions, extended-matching (R-type) questions were used with dichotomic scores. | Most students showed a problem-specific knowledge structure, and their diagnostic performance was superior to that of students with a generic structure (68.5 vs. 55.3%, d = 0.45, *p* = 0.004). |
| Da Silva and Dennick (2010)  United Kingdom | Observational explanatory | To demonstrate that an analysis of PBL sessions allows to enumerate and evaluate the technical vocabulary | First-year medical  students (*n* = 7) | **PBL** including three sessions.   - Identify key findings, explore additional data, synthesize the case and generate conclusions. | Measurement of the frequency of occurrence of words associated with the “subordinating conjunctions” category (i.e., if, then, when, and because), which are used to join a subordinate clause to a main clause | Statistically significant differences in word frequencies between different PBL sessions were detected. These differences can be related to the sessions’ focus (questioning vs. interpreting data). |
| Diogo et al. (2023)  Portugal | Single-center pilot | To analyze verbalizations during an OSCE and correlate them with test scores and final medical degree scores | Sixth-year medical students (*n* = 118) | **OSCEs**   - Realize a clinical history and a physical examination. - Complete a PEF. - Respond aloud to a one probing intervention that was integrated at the end of the OSCE. | Used an **institutional list of SQs**. “Strong” encapsulations encompass several “smaller” clinical concepts, whereas “weak” encapsulations might only represent simple word transformations into medical terminology. | Semantically “strong” verbalizations were longer and demonstrated more semantic transformations. Students who produced “strong” verbalizations displayed higher OSCE, case-based exam, and medical degree scores (*p* < 0.05) |
| Dore et al. (2012)  Canada | Quasi-experimental | To examine instantiated features in medical diagnosis | Psychology and health science students (1^st^ experimentation: 55; 2^nd^: 93; 3^rd^: 81; 4^th^: 34) | Case-based scenario  Identify all the features present in the case by typing them into one of the five boxes provided. Participants assigned probability to the four disorders. | Performed descriptive analysis of the probabilities assigned to disorders, represented by feature | Seeing a feature once in the appropriate context was sufficient to form a diagnostic association equivalent to instantiations seen four times in a different context. The impact of feature instantiation remained up to 24 h post-exposure. |
| Elieson and Papa (1994)  United States | Randomized controlled trial | To determine the effects of various knowledge formats on diagnostic accuracy | Third- and fourth-year medical students (*n* = 64) | **Resolution of fictitious computerized clinical cases**   - Diagnose 16 cases, where 18 features were identified.   Control group = no aid;  Aid A= Matrix; Aid B = Prototypical description  Aid C = Same as Aid A but with the information embedded in narrative form; Aid D = “hard” descriptors used in Aid C were transformed into “soft” text descriptors | The computer recorded the sequence of the participants’ keystrokes, and the time required to complete each case. | Aids A and B were better at helping to identify the distinguishing characteristics of the candidate diseases. The aids enabled students to identify the relevant between-category differences and within-category similarities. However, students using the matrix were able to rule in/out some diseases with a high degree of reliability. |
| Eva et al. (2010)  Canada & United States | Quasi-experimental | To test the extent to which the use of medicalese alters performance and evaluate the extent to which language difficulties and/or differences in medical knowledge cause(s) differences | International medical graduates (*n* = 1274, 38.6%);  Canadian medical graduates (*n* = 2025, 61.4%) | **Resolution of fictitious computerized clinical cases** (*n* = 6)   - List three diagnoses in order of likelihood. Features indicative of two diagnoses were presented uniformly in lay terms, medical terminology. and SQ, respectively, and in mixed combinations. | Responses were coded to determine whether candidates named the most plausible diagnoses in their individual differentials and whether those diagnoses were ranked first (i.e., were perceived by the candidate as being the most likely). | Uniform use of lay terminology resulted in the highest test reliability among the experimental conditions.  Medical and semantic terminologies have equal influence, and their use impacts diagnostic accuracy. |
| Guerrasio and Aagaard (2014)  United States | Prospective observational | To assess the effectiveness of a standardized CR remediation plan for medical learners at various stages of training | Medical students, residents, and fellows who had deficit and are in CR remediation (*n* = 151) | A 10-step remedial teaching plan, including the **resolution of fictitious written clinical cases**   - Develop a framework for creating a differential diagnosis based on SQ - List diagnoses in order of likelihood - Summarize cases using as many SQ in both written and oral format | Reassessment was performed post-remediation, including via OSCEs and the script concordance test, chart stimulated recall, etc. The results of the reassessments determined whether remediation was a success or a failure. | Prevalence of CR deficits did not differ by training level (*p* = 0.49). 96% passed the post-remediation reassessment. 72 % either graduated from their original program or continued to practice in good standing. |
| Hege et al. (2017)  Germany | Pilot | To design and implement a software tool to enhance VPs to foster the acquisition of CR and evaluate the tool’s usability and integration into the VP system | Fourth-year medical  students (*n* = 64) | **VPs** combined with a concept mapping tool   - Build patient’s illness script as a concept map. - Compose a short SS. | Use of **SSAR (Smith et al., 2016)’s rubric**. The score for the use of SQs in the SS was determined as follows: 0: comprising < 30% SQs; 1: comprising < 60% and ≥ 30% SQs; 2: comprising ≥ 60% SQs | Students submitted 65 final diagnoses, but only 36 connections were drawn, and 19 SSs were composed. The students interacted with the tool but created less concept map nodes and connections than the experts. |
| Hege et al. (2018)  Germany | Quasi-experimental | To explore the details of the CR process and assess undergraduate medical students’ diagnostic accuracy when working with VPs using a concept mapping tool | Undergraduate medical students (*n* = 317) | **VPs** combined with a concept mapping tool, including the composition of a short SS | The SS were scored based on the use of SQs. The unit of analysis was learners’ completed maps (i.e., having a final diagnosis) for a VP | Composing an SS with adequate use of SQs was found to be related to more nodes in all categories and higher scores on differential diagnoses and tests. |
| Heist et al. (2016)  Japan | Quasi experimental (prospective) | To explore trainees’ summary statement (SS) case summarization styles and ascertain VPs’ effectiveness at improving SS quality | First-year junior resident physicians from four residency programs (*n* = 54) | **Five VP modules**   - Complete a free-text SS | An **institutional scoring rubric for the SS** for each VP was used. Thematic analysis was used to identify SS styles. Coders evaluated SQs by identifying whether each SS was semantically driven. | From Modules 1 to 5, increases were observed in the use of a narrative summarization SS style (*p* = 0.016), the SS CR quality score (*p* = 0.021), and the percentage of semantically driven SSs (*p* = 0.003). |
| Longo et al. (2018)  United States | Pilot | To demonstrate the ability to use Bordage’s and Anderson’s methodologies to evaluate CR | Physician assistant students (*n* = 12) | **OSCEs**   - Write a SOAP note - Respond aloud to an interview (diagnosis) | TA interviews were coded using **Bordage’s discourse types and Anderson’s validated flow map analysis.** | Significant correlations were found between semantic, diagnostic, and knowledge network organization variables. Analysis of variance showed significant differences of knowledge network organization between weak vs. strong diagnosticians (*p =* 0.054). |
| McQuade et al. (2024)  United States | Observational | To elucidate characteristics that distinguish problem representation created by experts and  novices. | Internal medicine residents (*n* = 30) and inpatient teaching faculty (*n =* 54) | **Resolution of fictitious written clinical cases** (*n* = 2)   - Write a SS - Provide an unranked three‐item differential diagnosis for each case. | The degree of encapsulation: 0 represented repetition of information (e.g., “white  blood cells 14”), 1 represented renaming (e.g., “leukocytosis”), and 2 represented synthesizing features into larger concepts (e.g.,  combining fever, tachycardia, and infection into “sepsis”). | Regardless of expertise level, problem representations following a three‐part structure (e.g., demographics, temporal course, and clinical syndrome) and including  temporal SQs were associated with diagnostic accuracy (p < .01). |
| Mlika et al. (2023)  Tunisia | Randomized, Controlled, non-blind crossover trial | To compare SNAPPS and clinical reasoning technique in teaching CR | Third-year undergraduate medical students (*n* = 72) | **Resolution of fictitious written clinical cases**  One group: clinical reasoning technique – a collaborative-case presentation aloud  Other group: SNAPPS technique (TA) | Use of the **NICTALOP** method comprising assessment of criteria: NI: the number of key ideas or key diagnoses; CT: the choice of terms; A: the veracity of the concepts or diagnoses; and LoP: the length and position of the ideas and concepts. | No significant statistical difference was observed between the mean scores according to the method used (SNAPPS vs. clinical reasoning technique; *p* = 0.890). |
| Nendaz and Bordage (2002)  United States | Randomized controlled trial | To measure the effect of an instructional intervention on diagnostic  argumentation and diagnostic accuracy during case write-ups | Second-year medical students (*n* = 60)  Intervention group (*n* = 20)  Control group (*n* = 40) | Workshops incorporating videotapes of students’ **examinations of SP**   - Elicit the basic attributes of an SP complaint - Summarize the case - Use the summary to compare/contrast diagnoses - Complete the patient findings questionnaire (MQC) - recall findings gathered during the SP encounter | Use of the **data collection checklist,** a **PEF, case summaries**, and **write-ups**. Assessment of diagnostic accuracy: 1 = accurate, 2 = parts of a differential diagnosis, 3 = related but vague diagnosis, and 4 = unlikely diagnoses. Use of SQs ascertained by counting the proportion of arguments to contrasted diagnoses using SQs | The intervention group used significantly more SQs in their summaries than the control group (*p* = 0.006). The correlation between checklist and PEF scores was higher for the intervention group than for the control group (*r* =0.70 vs. *r* = 0.58, *p* > 0.001). No differences were found in the number of SQs used in write-ups or in diagnostic accuracy (*p* > 0.56). |
| Patel et al. (2005)  Canada | Quasi-experimental | To investigate the effect of curricular change on knowledge integration and reasoning processes during problem-solving | Medical students from three levels of training (*n* = 18) | **Resolution of fictitious written clinical cases**   - Provide diagnostic explanations before and after exposure to basic science information | **Propositional and semantic analysis** according to the links between the propositions labeled and the types of relations, i.e., causal relationship, conditional or if/then relationship, resulting relations, location and proximity relations | Students generated fewer inferences and used more information from the basic science text. They generated more elaborations during explanations using a mixture of data- and hypothesis-driven strategies. |
| Schaye et al. (2019)  United States | Quasi-experimental | To test whether a CR curriculum  would improve the diagnostic reasoning process and the application of core concepts taught in the curriculum | First-year medical residents (*n* = 71) | **Resolution of fictitious written clinical cases and encounter with patients**  Group 1: no intervention; Group 2: Part 1 = Iterative resolution of written clinical cases; Group 3: (Parts 1 & 2) Part 2 embedded within an inpatient clinical rotation  Meeting with a senior   - Summarize aloud findings and state a diagnosis and a management plan. | Bordage et al.’s [36] **diagnostic thinking inventory self-assessment** was used to assess the approach to diagnostic reasoning. | Significant differences were found in the application of concepts [no intervention 1.6 (0.65) compared to partial 2.3 (0.81) and full 2.2 (0.91), *p* = 0.05] as well as in describing cases in problem representation format [no intervention 1.2 (0.38) and partial 1.5 (0.55) compared to full 2.1 (0.93), *p* = 0.004]. |
| Smith et al. (2014)  United States | Descriptive cohort | To study the linguistic and non-linguistic elements of diagnostic reasoning across the continuum of medical education | Premedical  students, medical students, residents, and experienced faculty (*n* = 50) | **Semi-structured interviews** with trigger questions   - Diagnose three common causes of dyspnea. | **Propositional analysis and concept mapping** were used to explore rational diagnosis and grounded theory, and a measure of emotional tone was used to explore aspects of intuitive diagnosis. | Semantic memory increased with experience, particularly for the recall of prototypical examples used to make a diagnosis. Concept maps increased in complexity with ascending education levels from first-year students to residents. |
| Wolpaw et al. (2009)  United States | Randomized controlled trial | To examine whether a structured case presentation technique (i.e., SNAPPS) can facilitate the expression of diagnostic reasoning and uncertainties in an actual office setting | Third-year medical students (*n* = 64). | **Encounter with real patients**  **Intervention group: learning SNAPPS** (via DVD, roleplaying, etc.)  Control groups: Feedback training (controlling for training time) and usual and customary instruction   - Audiotape case presentations | Presentation content was coded according to length; conciseness, summary thoroughness, basic attributes, completeness, number of diagnoses in the differential, etc. | Students using the SNAPPS technique produced more concise summaries than students in both the comparison and usual and customary groups (0.48 compared with 0.62 and 0.62, *p* < 0.000). |

**CR**: Clinical reasoning; **IDEA**: Interpretive summary, Differential diagnosis, Explanation of reasoning, and Alternatives; **NICTALOP:** NI: number of key ideas; CT: choice of terms; A: the veracity of the concepts/diagnoses; and LoP: the length and position of the ideas and concepts.: **OSCE**: Objective Structured Clinical Examination; **PBL**: problem-based-learning; **PEF**: postencounter form**;** **SNAPPS**: Summarize history and findings, Narrow the differential, Analyze the differential, Probe preceptor about uncertainties, Plan management, Select case-related issues for self-study; **SOAP:** Subjective, Objective, Assessment, Plan; **SQ**: semantic qualifier; **SP**: standardized patient**; SS**: summary statement; **TA**: think aloud; **VP**: virtual patients.
