# Supplementary material for: Transformation and articulation of clinical data to understand students’ clinical reasoning: a scoping review
Source: BMC Med Educ. 2025 Jan 12;25:52. doi: 10.1186/s12909-025-06644-7 (PMC11725190; doi:10.1186/s12909-025-06644-7)
Supplement: Supplementary file 4 — Supplementary Material 4 [file 12909_2025_6644_MOESM4_ESM.docx]

**Appendix D. Characteristics of studies related to the development/use of tools or rubrics**

| Authorship, country | Study type | Aims | Population | Tool or rubric developed/used | Training context/Assessing CR |
| --- | --- | --- | --- | --- | --- |
| Baker et al. (2015)  United States | Descriptive | To assess students’ CR based on written notes and present validity evidence in support of the IDEA assessment tool | Data from 216 new patient admission notes compiled by 128 students | The **IDEA instrument** | Students summarized key features of a patient’s history, physical examination findings, and initial exam results using SQs then compiled a short list of the most likely diagnostic possibilities and committed to one diagnosis as being the most likely. |
| Bordage et al. (1997)  United States | Methodological | To establish the validity of the semantic classification of discourse to previous measures of clinical competence | Third-year medical students (*n* = 19) and expert in rheumatology (*n* = 4) | **Bordage’s simplified semantic classification method** | Students and rheumatologists examined two SP and did a case presentation aloud. Presentations were transcribed and coded using a 3-level classification: 0 = few or no transformations, l = some transformations but none used to contrast the diagnostic possibilities, and 2 = purposeful transformations into SQs to sort two or more diagnoses. |
| Cianciolo et al. (2021)  United States | Single institution  pilot | To explore the potential of using ML technologies to score one such assessment, namely the diagnostic justification essay | 414 medical students (*n* = 700 essays) | **A machine scoring system** | Using simulated patients, students wrote a diagnostic justification essay. The machine scoring system incorporates several semantics based on NLP metrics to assess the quality of the summative clinical competency exam including the diagnostic justification essay. Machine scores ranged from 1 (*poor*) to 4 (*excellent*) for each criterion (i.e., differential diagnosis, recognition and use of findings, workup, and thought process). |
| Carter et al. (2018)  United States | Methodological | To assess the validity of the PBEAR tool | A panel of pediatric  clerkship students (*n* = 53) | **The PBEAR tool** | Completion of an OCP during inpatient rounds or for assessment (with reference to OCP records after rounds). |
| Covin et al. (2020)  United States | Descriptive correlational | To determine the convergent validity of three CR instruments: the **CRT** checklist, the **PNS** rubric, and the **SSAR** by comparing each instrument’s scoring of clinical notes | Medical students (*n* = 235) | **CRT (Goldszmidt et al., 2013)**  **PNS (Park et al., 2013)**  **SSAR (Smith et al., 2016)** | Students worked in small groups on a computer-based case presentation. The case paused twice for students to input a working differential diagnosis and plan. At the conclusion, each student wrote an individual clinical note. Students were randomly selected to write a clinical note. Each note was scored with CRT, PNS, and SSAR. Final scores were compared to Clinical Data Interpretation test. |
| Dory et al. (2016)  Canada | Methodological | To provide evidence of the validity of written case summaries as assessments of clinical problem representation | Fourth- and fifth-year medical students (five of whom were in Year 5) (*n* = 700) | **An institutional scoring rubric** | Examinations comprised eight patient vignettes using lay terminology. Students summarized the case in a few sentences. The scoring rubric had three criteria: extraction of pertinent findings, semantic quality, and global criterion. |
| Durning et al. (2012)  United States | Methodological | To explore the feasibility, reliability, and validity evidence for using a PEF in assessing CR. | Medical students  (*n* = 170) | **PEF** | A free-text PEF was used at an OSCE station. Scores from individual items on the PEF and other components of the OSCE station (i.e., SP’s checklist and oral presentation rating form) were compared. Scores were also compared with other course exam grades. |
| Fleiszer et al. (2018)  Canada | Descriptive | To determine whether student components of online patient management can be used to measure changes in CR | Medical students comprising clerkship juniors (*n* = 57) and seniors (*n* = 25) | **Institutional procedural and semantic scoring rubrics** | Students summarized the case using an SS consisting of free-text input. SSs were scored using both rubrics. One point was allocated to each element addressed in the SS. The semantic rubric requires specific phrasing in the SS |
| Hege et al. (2020)  Germany | Pilot | To combine **SSAR (Smith et al., 2016)**’s rubric with NLP approaches to test whether automatic assessment of SSs can serve as a basis for providing automated feedback | Worldwide health care students (*n* = 100) | **SSAR (Smith et al., 2016)** | Five VPs were provided in two open-access courses where learners composed a short SS that was analyzed based on **SSAR (Smith et al., 2016)**’s rubric, plus an additional category (VP’s name). An NLP approach was implemented to automatically assess 125 randomly selected SSs, and the manual and automatic ratings were compared. |
| Park et al. (2013)  United States | Methodological | To examine the validity evidence for the PNR | Fourth-year medical students (*n* = 170) | **PNS** | The PNS was developed for an exam to assess written patient notes. The rubric measures three dimensions: documentation, justified differential diagnosis, and workup. Each examinee participates in 12 encounters with SPs. After each encounter, the examinee writes a patient note. |
| Schaye et al. (2021)  United States | Methodological | To develop a valid and reliable assessment tool for CR documentation by building on the IDEA assessment tool | Notes from medical residents and subspecialty fellows (*n* = 252) | **The revised IDEA assessment tool** | The IDEA assessment tool was used to review written admission notes. The revised IDEA assessment tool was created through discussion. Its validity was assessed by rating 252 notes. |
| Smith et al. (2016)  United States | Methodological | To describe the development and preliminary validation of a rubric to assess SSs | MedU student users created 83,347 SSs for online VP cases. 120 SSs were randomly selected. | **SSAR (Smith et al., 2016)** | To create the rubric, researchers coded 50 SSs from a large database of medical students’ SSs from VP cases. Through an iterative process, they reached a consensus on an assessment rubric and applied it to 60 additional SSs. |
| Torre et al. (2021)  United States | Methodological | To gather validity evidence for the currently used MSX format by exploring the relationship between MSX scores and other scores on external measures of CR abilities | Medical students (*n* = 477) | **Evolving MSX** | Students watched a video of a patient interview and answered related questions. MSX Step 1 comprises descriptive, differentiating, and contextual items. Step 2 comprises the problem list, reporting facts, and using SQs. Step 3 concerns the aptness of the diagnosis, which entails supporting and refuting items, communicating the major reason for the diagnosis, and drawing a conclusion given the likelihood of the listed diagnosis. |

**CR**: Clinical reasoning; **CRT**: Clinical reasoning task; **IDEA**: Interpretive summary, Differential diagnosis, Explanation of reasoning, and Alternatives; **H&P :** History and Physical; **MCQ**: multiple choice questions; **ML**: machine learning; **MSX**: multistep exam; **NLP**: natural language processing; **OCP**: oral case presentation; **OSCE**: Objective Structured Clinical Examination; **PBEAR**: Problem Representation, Background Evidence, Analysis, Recommendation; **PEF**: postencounter form; **PNS**: Patient Note Scoring; **SP**: standardized patient; **SQ**: semantic qualifier; **SS**: summary statement; **SSAR**: summary statement assessment rubric; **VP**: virtual patient
